# Asylums for the Insane

**Published:** 1848-09

**Authors:** 


### EDITORIAL DEPARTMENT.

*Asylums for the Insane.*—The progress of Civilization is, perhaps, in no department of philanthropic effort more conspicuously displayed, than in measures adopted for the relief of our fellow beings afflicted with that most terrible of all dispensations—insanity. It is not long since society recognized no other duty with regard to these unfortunates, than its own tranquility and protection; and, so that they were prevented from annoying or injuring others, their condition was considered of but little moment. With this single aim, they were confined, frequently secured by chains, and in every respect treated with less consideration than those by whose rational, voluntary misdeeds, the laws of society had been infringed. The situation of the felon was far more comfortable than that of the lunatic. The name of *mad house,* was associated with deprivations and cruelties greater than were attached to the idea of the jail or prison; so that if a sane man, half a century ago, had been compelled to elect between Newgate or Bedlam, (names which are classical in English literature,) he would have had no hesitancy in preferring the former to the latter.

A new era in the management of the insane may be dated from the time the illustrious Pinel removed the fetters from the madmen within the walls of the *Bicetre,* of Paris, Then commenced the recognition of the claims of this unfortunate class upon society for comfortable accommodations, humane treatment, and measures better calculated to effect restorement. On comparing the well-regulated Asylums of the present day, with the mad houses of former days, the contrast is so great, that it may with propriety be said to denote a revolution, rather than a reformation. Our country has not been backward in this field of philanthropy. Some of our institutions are probably not excelled by those of any country. All that talent, experience and zeal, aided by ample pecuniary resources, can accomplish, appears to be realized in the more distinguished of the Insane Asylums of the United States. We are by no means, however, justified in asserting that the requisitions of humanity with respect to the Insane, are universally and completely fulfilled in our country. In some sections, Asylums have never been established, and in other sections they are inadequate, in number, or size. Many Institutions are compelled to reject the incurably insane, their accommodations being insufficient, if they are received, for those cases in which a cure may be hoped for, and the latter, under such circumstances, very properly having precedence. It is needless to say that the former should be provided for. There are, as we learn from an editorial article in the last number of the Journal of Insanity, published at Utica, N. Y., about thirty Institutions in the United States. This embraces fifteen State Institutions, the remainder being made up of corporate Institutions established by philanthropic individuals, private Asylums, and departments for the Insane connected with alms houses, or general hospitals. These Institutions are, nearly all of them, full; and the aggregate number of inmates amounts to 4,711. In so far as statistics authorize a calculation of the number of Insane persons in the United States, exclusive of Idiots, the above number of patients already provided for, falls short of one half of the number of those who claim the advantages of public Institutions Constant progress, as we learn from the article just referred to, is being made in the establishment of new Institutions, and in extending the accommodations of those already established. In Illinois, Louisiana and Missouri, State Institutions are soon to go into operation. Pennsylvania and Tennessee have also recently appropriated large sums for this object. The time must arrive, and, as we trust, ere long, when the absence or insufficiency of Asylums will furnish no excuse for this class of unfortunates being confined, as heretofore, (and now to some extent), in cellars, outhouses and jails, in any section of our country.

The establishment of Asylums is, of course, the first point. Of their propriety and usefulness there can be no question. As regards their construction, and organization, there may be many points which admit of discussion. We do not, however, propose, nor are we prepared to engage in any inquiries, at present, concerning these points. We would simply remark, that there seems to be a tendency to enlarge Insane Establishments to an extent, which, as it seems to us, cannot but compromise, in no small degree, their usefulness, We will not pretend to state any exact limitations of magnitude which should be adhered to in all instances, but, it seems to us very clear, that the individualities of each case cannot be so well studied, and managed, by the Physician of an Institution embracing several hundred patients, as if his attention were divided among a fewer number of cases. It is true, the Physician may be aided in his duties by assistants, but the latter are generally young, and comparatively inexperienced, and it may rationally be claimed, that every patient shall have the benefit of the abilities of the Physician in chief, whose position implies peculiar qualifications. In most Institutions the Physician is also general Superintendent of the Establishment. This imposes a multiplicity of duties not strictly medical, which, if the Institution be very extensive, must necessarily encroach on the time and thought, which it were better should be exclusively devoted to the mental and physical condition of the patients. While it undoubtedly is important that the medical officer should be empowered to exercise complete control over all the affairs of the Institution, it is also important that he should be relieved, as much as possible, of all drudgery disconnected from his professional duties.

The perfection of the medical and moral management of the Insane, we imagine, can only be realized in private retreats, where the number of patients is very few, and the Physician can more effectually investigate the peculiarities of each case, and adapt his remedies and his discipline accordingly. Few, however, can afford to pay for such exclusive attentions, and, hence, for the mass of Insane, public Asylums are indispensible.

The most prominent of modern measures of relief for the insane, are the greater number, and the greater commodiousness of institutions for their reception. But it is in the principles upon which these institutions are conducted—in other words, the principles upon which insanity is treated, that we find the most striking evidences of that contrast, which, as we have said, denotes a revolution in this province of medical philanthropy. As the more important of the changes referred to, without dwelling upon the subject, we may enumerate:—the abolition of all harsh measures, such as seclusion, corporeal punishment, restraint, or coercive measures, substituting therefor, indulgence of personal liberty within certain limits, and uniform kindness in the intercourse between the patients and their attendants; mental and physical occupation in agricultural and mechanical employments, and, to a certain extent, in intellectual and educational pursuits; amusements; religious influences—these relate to the principles of moral management. As respects strictly medical treatment, the difference between the present and past appears to consist, not so much in the application of new remedies, or principles of therapeutics, as in the more sparing use of powerful agents, or, in other words, a nicer discrimination in their employment. In the latter point of view, the improvement in the treatment of insanity, corresponds with the improvement in therapeutics as applicable to diseases generally.

Much interesting and useful information is communicated to the profession, and the public, concerning the treatment of insanity, and the various subjects entering into its pathology, ætiology and history, by means of the annual reports emanating from the different institutions of our country.

Our design in commencing this article, was to notice, briefly, some of these reports which have been received by us within the last few months. By indulging in some prefatory remarks, we have been betrayed already into nearly as many words as we had intended the whole article should have embraced. The objects of these reports are, in the first place, to give an account of the particular institutions from which they emanate, and, next, to contribute statistical, and other facts relating to any, and all of the subjects appertaining to insanity. A collection of them extending over a series of years, must contain data for valuable deductions. We should be glad to see a similar plan generally adopted by the medical officers of general hospitals, alms-houses, &c. In this way, as it seems to us, our medical literature would be enriched by periodical contributions, the continuance of which, and also their constant improvement, would be likely to be secured by a healthful spirit of emulation.

The substance of the reports which we shall proceed to notice, cannot be presented, however much condensed, without occupying more space than it would be proper to devote to this collateral branch of medical science. Such a synopsis is the less called for, from the fact that the reports themselves have a pretty extended circulation, and are probably in the hands of many of our readers. We shall content ourselves with giving a very brief summary of some of the more prominent items of intelligence appertaining to each institution.

We take up first, the "*Fifth Annual Report of the Managers of the State Lunatic Asylum,*" *at Utica, N. Y.* The capacity of this asylum will appear from the following extracts from the report:—

"The main edifice, the erection of which was commenced in 1839, and finished in 1842, is 550 feet in length, the centre part of 120 feet front, four stories high, and the wings three stories, exclusive of the basement.

"It is built of hewn stone, in the Doric style of architecture, the portico in front being supported by six fluted columns, built of blocks of the same stone, eight feet diameter at the base, and forty-eight feet in height.

"Connected with, and in rear of the main building are two buildings of brick, three stories high, exclusive of the basement, extending back at right angles with the wings of the front building, two hundred and forty feet, and the rear of these buildings is connected by two buildings parallel with the front, one hundred and forty-six feet each, twenty-five feet wide and two stories high, thus forming an enclosed yard in the rear 215 by 292 feet.

"There are in all, 380 single rooms for patients, 24 for their attendants, 20 associated dormitories that will accommodate from five to twelve persons each, 16 parlors or day rooms, 12 rooms for dining, 24 for bathing, and as many for clothes, and the same number for water closets."

The price charged for patients supported by towns or counties is $2 per week, and for patients supported by their own property, or by their friends, $2,50 to $4 per week.

As medical men are often applied to for information respecting the course required for transferring patients in indigent circumstances to the asylum, we quote the following extract, containing the statute on this subject, for the convenience of those of our readers who reside in this state:—

"By the 26th section of the act to organize the asylum, (chap. 135 of laws of 1842,) it is provided that when a person in indigent circumstances, not a pauper, *becomes* insane, application may be made in his behalf to the first judge of the county where he resides, and the said judge shall call two respectable physicians as witnesses, and fully investigate the facts of the case, and if the judge certifies that satisfactory proof has been adduced showing him insane, and his estate insufficient to support him under the visitation of insanity, he shall be admitted into the asylum and supported there at the expense of the county, until he shall be restored to soundness of mind, if effected in two years."

The number of inmates (patients) in this institution, for the year ending November 30th, 1847, was 802. Of this number, 428 were admitted during the year, the balance remaining at the close of the preceding year. The superintendent states, that the number admitted during the year is greater than were "ever received in one year into any other institution exclusively devoted to the reception of the insane of both sexes."

The superintendent advises not to remove patients to the asylum too early after the first manifestations of insanity. It may prove a transient affection, and at its early stage, is apt to be connected with conditions of the system which are aggravated by the journey to the asylum. He states that patients are sometimes brought to the asylum, suffering from inflammation of the brain, which had been mistaken for mere insanity. He remarks that bleeding in cases of insanity is "generally improper, and frequently very injurious."

The medical officers of this institution consist of the superintendent, two assistant physicians, and two apothecaries, the latter being either Physicians or Students of medicine. Dr. Amariah Brigham, the superintendent, is well known to all our readers as a gentleman eminently fitted for the responsible position which he occupies.

It will readily be conceived that the duties which devolve upon the head of such a mammoth institution must be immense, and could hardly fail to prove too onerous for health, as has been the case. We learn by a late number of the Journal of Insanity, that Dr. B. has recently devoted a few weeks to travelling, by which we trust, his health has been restored

We need not add, after what has been already remarked, that we doubt the policy of gathering so many patients in one institution. A proposition to establish an asylum in the western portion of the State, has been for two or three years before the Legislature, and we trust it will soon be acted upon. By drawing away a portion of the inmates of the Utica asylum, the usefulness of the latter institution would be enhanced, and further accommodations for lunatics (which are needed) afforded, which would be more accessible to patients in Western New York.

Dr. Brigham, in addition to his other duties, is editor of the "American Journal of Insanity," a quarterly publication, printed at the asylum, for which every practitioner of medicine would do well to subscribe. We believe this is the first, and only periodical in the English tongue devoted to the subject of insanity.

"*Twenty-seventh Annual Report of the Bloomingdale Asylum for the Insane.* By Pliny Earle, M. D., Physician to the Asylum."—The number of persons admitted into this institution during the year, was 143. Thirteen of these were cases of delirium tremens and habitual inebriety, leaving, as cases of insanity proper, 130. At the commencement of the year there were 131 patients, at its close there were 145. Of 116 patients discharged during the year, 58 were cured, 17 much improved, 23 improved, and 18 unimproved. The number of deaths was thirteen. The moral treatment here, as in other modern well regulated establishments, embraces manual labor, religious worship, recreative exercises, amusements, and instructions by means of schools, lectures and library.

The report contains a meteorological register for the year. We have also received a pamphlet of 136 pages entitled "*History, Description and Statistics of the Bloomingdale Asylum for the Insane.* By Pliny Earle, M. D., etc.," which, to those interested in researches connected with the subject of insanity, must prove a highly interesting and valuable publication. After giving an account of the rise and progress of the Asylum, and the principles upon which it is conducted, Dr. Earle submits results of an analysis of the cases from 1821 to 1844, as regards a variety of points connected with the subject of insanity. Dr. Earle is zealously devoted to scientific investigations bearing on this subject, and in addition to this memoir, has contributed several valuable papers, of late, for different medical periodicals.

The Bloomingdale Asylum is a branch of the New York Hospital. It was originally sustained by private benevolence, but for many years, as now, is supported chiefly by the State. It is located within the limits of the municipal jurisdiction of the city of New York, near the banks of the Hudson river, and embraces a farm of fifty-five acres.

In addition to the asylums at Utica and Bloomingdale, the State of New York contains four institutions for the insane. The New York City Asylum, at Blackwell's Island, is a city institution, and receives pauper lunatics from the city of New York. The condition of this asylum was severely commented upon by the convention of Superintendents of insane asylums recently assembled at New York. We learn that measures are in progress for its improvement. This asylum contained in August, 1847, 417 patients.

Queen's County (Flat Bush) Asylum contained, at the same date, 70 patients. We presume this is a county institution. The remaining asylums are private institutions, one situated at Hudson, N. Y., under charge of Dr. White, containing, at the same date as above, 20 patients; the other, under charge of Dr. Macdonald, situated at, or near New York, containing 30 patients.

"Thus," to quote the language of Dr. Earle, "more than one thousand one hundred of the insane are now provided for at the institutions within the State; and, yet, the wants of the community are not fully supplied."

*Fifteenth Annual Report of the Trustees of the State Lunatic Hospital at Worcester, Massachusetts, Dec.*, 1847.—In this report the history of every case is exhibited in a tabular form, showing age, sex, civil condition, supposed cause, time of admission and discharge, etc.

The institution has provided for 607 patients during the year. Of this number, 367 were remaining at the close of preceding year, and 394 were remaining at the date of this report. 240 were admitted during the year. Of the 213 who left the hospital during the year, "103 had recovered, and were able, on their return to their homes, to renew their former duties of life, and station in society. Twenty three were convalescent when they were taken away, some of whom, we believe, have since fully recovered. Pecuniary considerations have led towns and individuals to procure the removal of forty three, most of whom would probably not have recovered, had they remained. Thirty have died.

"The hospital has been in a crowded condition every day for the past year.

The established rate for board is two dollars and fifty cents per week."

We quote the following paragraph of the report:—

"Massachusetts has done more for its citizens, in this department of benevolence, than any other portion of this country. More than one in a thousand of her population are provided for, in public and private hospitals for the insane. It is safe to predict, that an accurate census of the insane and idiots, who are incapable of taking care of themselves, would show that they were as many as four to every thousand of the inhabitants of the Commonwealth. And such, too, I believe to be the melancholy fact in regard to their proportion in the neighboring states.'

This report contains a register of the weather for the year.

Dr. Chandler, the able superintendent, recommends that patients be not brought to the Asylum earlier than the third or fourth week from the commencement of the insane attack, in order to avoid the introduction of cases of disease in which insanity is only one of the symptoms, which, in such cases, often subsides when the disease terminates. He states that "six months is about the average time that it takes patients to recover from insanity."

"*Report of the Eastern Asylum in the city of Williamsburgh, Virginia,* 1847."—This report embraces a period of nine months from Jan. 1847. The number of patients on the 12th of January was 140. During the period of nine months, forty-three patients were admitted. The number of discharges is 12, all except two being cases of recovery. Seven deaths occurred. Number of patients at the time of the report, 164.

Dr. John M. Galt, author of a learned work on insanity, is superintendent of this Institution. Like most other asylums this is crowded, and an increase in its capacity is asked for. Dr. G. discusses the advantages and disadvantages of large institutions, and expresses an opinion adverse to the policy of making them very large. He thinks, however, the accommodations at the Eastern Asylum may be enlarged to some extent, without impairing its usefulness.

Dr. Galt's experience is opposed to bleeding in insanity. We quote the following paragraph relating to that point:

"We observe by the papers accompanying patients when brought to this institution, that bleeding is still a measure employed in insanity, by some physicians of Virginia. In the patients received here, depletion of any kind seems rarely, if ever requisite. The medical attendant of one of the cases admitted recently, remarks in his testimony at the time of her committal, that blood-letting was employed but once, and "with very apparent disadvantage." In numerous instances on the other hand, a tonic or strengthening mode of treatment is evidently demanded. Perhaps of all medicines administered here with this end, most use is made of the Thoroughwort, (Fupatorium perfoliatum) an indigenous plant. We have large quantities of it gathered every year."

"*The Fifth Annual Report of the Mount Hope Institution near Baltimore, for the year* 1847. By William H. Stokes, M. D.—This report contains a register exhibiting the general history of each patient. The whole number of patients in the asylum during the year, was 128. Of these, 56 were remaining at the close of the preceding year, 72 admitted, and 54 remaining at the time of the report.

Cases of *mania a potu* are excluded from the foregoing enumeration.

Of the 72 cases discharged during the year, 35 were recovered, 27 were prematurely removed, contrary to the advice. and without the consent of the physician, 3 eloped, 7 died. Dr. Stokes comments upon the evils of premature removals from asylums, a subject on which the experience of all superintendents is united. The per centage of recoveries in this institution is very large, (71·4 per cent on average number,) and the per centage of deaths is also extremely low, (2 per cent on average number.) Dr. Stokes adduces the average per centage of recoveries, and deaths, of fifteen asylums, Mount Hope taking precedence in each. This speaks well for the Mount Hope Asylum, although there is much truth in the following remark by Dr. Brigham: "The management of an establishment for the insane where but few are annually received, and those few for the most part incurable, is necessarily different from that required where the reception of new cases is very large. *Hence the uselessness of comparing results of treatment.*"

Dr. Stokes attributes the superior success of the treatment of insanity in the Mount Hope Asylum to the efforts of the Sisters of Charity, by whom, in connection with superintendent, the institution is conducted. We quote his remarks on this subject:

"In an establishment for the insane every regulation, every action, the spirit of every remark, almost every look may be invested with remedial power, or be pregnant with mischief. In fact nothing is trifling, nothing is wholly destitute of effect, for good or evil, in a house full of infirm and irritable minds. The deportment of those in habitual attendance upon them, their manner, their tone of voice, may all sensibly affect the sensitive organizations for the relief of which the whole institution is designed. High as we regard medical treatment, we still feel satisfied, that kind looks, and little acts of attention, denoting a real sympathy for them, are often more efficacious than medical prescriptions, The latter without the former must prove unavailing. Henee we believe that the greatest amount of good is achieved, not in institutions presenting the most imposing and expensive architectural arrangements, or in which heroic remedies, and strong impressions are trusted to, but in those in which, with mild medical treatment, are brought to bear the continual influences of a benign and paternal authority, a profound knowledge of the human heart, and a philanthrophy which finds its greatest happiness in alleviating human suffering. From these remarks, it will be seen, how much of remedial power and influence may be wielded by those having the daily and hourly charge of the insane. The task of administering to disorders which distract the reason, and pervert the affections, and afflict the fancy with false or fearful images, is of a highly delicate character—too delicate to be intrusted to others, than to those whose thoughts and mental habits have subjected them to a long preparation for it. In the Sisters of Charity are associated a combination of qualities, peculiarly and fully adapting them for these important duties. The high per-centage of recoveries in this institution is no doubt mainly attributable to their untiring assiduity and devotion, to their perfect self-abandonment, and self-sacrificing zeal in the discharge of the duties committed to them. Deeply imbued, as their hearts are, with those principles and feelings, which are the direct emanations and blessed fruits of that enlightened and universal *charity*, which has its imperishable root in the Christian religion, they also bring to bear those influences, which female ingenuity, and womanly tenderness can alone devise and apply. Man's less delicate sensibility can scarcely comprehend, much less employ the ready resources she ever has at hand to encourage and soothe the patient. She best knows how to reanimate the feminine heart by such confidential employments, and how to influence it by such arguments and suggestions as man's more difficult nature can never become master of.

But it is at *the period of convalescence* especially, that the zeal and uncalculating philanthrophy of the Sisters prove in a high degree auxiliary to the efforts of the physician. As the atmosphere of the mind begins to clear, the recovery of the patient is greatly promoted, by having the assistance of those possessing sufficient intelligence and tact, to steady the faltering reason, and to aid in dispelling the dark clouds which, still to an extent, overshadow the mental faculties. Brought, as the Sister is, into constant and familiar contact with the patient, she is the first to discover the early dawnings of returning reason, and much now depends upon the manner in which the mental powers are assisted in emancipating themselves from the morbid bondage. If any lingering delusion is, at this critical moment, harshly opposed, or any painful recollections suddenly revived, much injury may be done to the patient, and his convalescence be greatly retarded. If, on the other hand, his feelings are sought to be calmed by suggestions advanced with kindness and sincerity, and in the soothing language of friendship—if the early glimmerings of reason are elicited by gentleness and kindness, and strengthened by the voice of comfort and encouragement, the healthy balance of the mind will speedily be restored, and reason again resume its throne. This happy faculty, of managing aright the important period of convalescence, is the reward alone of prolonged intercourse with the insane and devoted attention to them; and therein consists the distinguishing and crowning advantage of this institution."

Dr. S. enforces the importance of an early removal of patients to an asylum in the following:—

"Thus of the aggregate number of one hundred and thirty four recent cases dicharged in five years, one hundred were restored, thirty-two prematurely recovered, and two died. This result will be conceded by every one, not only to reflect credit upon the institution, but to demonstrate, incontestibly, the ready curability of almost all cases subjected to early treatment. As high a percentage of cures can hardly be predicted of any other malady to which the human system is liable. With these facts before us, we unhesitatingly avow it as our firm conviction, that if patients were placed under favorable circumstances for recovery, whilst the disease is in its acute stage, it would soon become a rare spectacle to witness a case of hopeless, incurable lunacy, within the walls of our asylums. Stronger evidence in corroboration of this fact could not be furnished than the following—*but three of all the cases admitted as recent during a period of more than five years have terminated in dementia, and remain inmates of the house in a chronic, incurable state of mental disease.* Many of the recent cases admitted during these five years were, unfortunately, prematurely removed by friends, and on their own responsibility. How the malady issued in those cases we are unable to say. But all the acute cases, that were fully and unconditionally consigned to our care, with the determination to abide the issue, have been restored to mental health, and to the enjoyment of their mental faculties, *with the exception of five.* Of this number two died, and *three* remain with us laboring under chronic insanity, with little or no prospect of a restoration. Hence we say, that *would an immediate resort be had in all cases, to active measures of relief, whilst the disease is still recent, cases of hopeless, remediless lunacy would henceforth be rarely witnessed within the walls nf our asylum.*

"*Report of the Chicago Retreat for the Insane.* By Edward Mead, M. D., Superintendent."—The legislature of Illinois at its last session, enacted a bill for the establishment of a State Lunatic Asylum. Two years, however, will be required for the erection of a suitable edifice. In the mean time, Dr. Mead, who has bestowed much attention to the subject of insanity, and who has been for several years past zealously occupied in bringing the importance of state provisions before the people, and the legislature, has opened a Retreat at Chicago as a private enterprize. This report is the first he has issued. It is dated February, 1848, and the institution had been in operation since August, 1847. Fifteen patients had been admitted, five of whom were restored, one had died of hisease of heart and lungs, and nine were remaining at the Retreat. Dr. Mead's accomodations are at present limited. The Retreat, however, is intended to be permanent, and an edifice is now under construction, situated two miles from Chicago, near the lake shore, which will accommodate, when completed, 150 patients. We hope that the result of the Dr's undertaking will prove profitable, both to himself, and those who may avail themselves of the benefits of his institution.

This completes the list of reports with which we have been favored. We are fully aware that we have done them but feeble justice in the meagre notices which we have given, but the facts which they contain cannot well be presented more concisely than they are in the reports, and to do them full justice, it would be necessary to transfer them entire to our pages. The profession generally cannot but take a deep interest in the subject of insanity, and it is a source of satisfaction, and pride, that this province of medical knowledge, and humanity, is being cultivated in this country with a degree of zeal and success, which, in the end, cannot fail of effecting all that science, and the most enlightened philanthropy can reasonably expect to accomplish.

